# Aquaporins in Brain Edema and Neuropathological Conditions

**DOI:** 10.3390/ijms18010055

**Published:** 2016-12-28

**Authors:** Aristotelis S. Filippidis, Richard B. Carozza, Harold L. Rekate

**Affiliations:** 1 Division of Neurosurgery, Beth Israel Deaconess Medical School, Harvard Medical School, Boston, MA 02115, USA; 2 Department of Neurosurgery, Boston Medical Center, Boston University, Boston, MA 02215, USA; 3 Boston University School of Medicine, Boston, MA 02118, USA; rcarozza@bu.edu; 4 Department of Neurosurgery, The Chiari Institute, Hofstra Northwell School of Medicine, Hempstead, NY 11549, USA; HRekate@northwell.edu

**Keywords:** aquaporin 1 (AQP1), AQP4, aquaporins, neuroscience

## Abstract

The aquaporin (AQP) family of water channels are a group of small, membrane-spanning proteins that are vital for the rapid transport of water across the plasma membrane. These proteins are widely expressed, from tissues such as the renal epithelium and erythrocytes to the various cells of the central nervous system. This review will elucidate the basic structure and distribution of aquaporins and discuss the role of aquaporins in various neuropathologies. AQP1 and AQP4, the two primary aquaporin molecules of the central nervous system, regulate brain water and CSF movement and contribute to cytotoxic and vasogenic edema, where they control the size of the intracellular and extracellular fluid volumes, respectively. AQP4 expression is vital to the cellular migration and angiogenesis at the heart of tumor growth; AQP4 is central to dysfunctions in glutamate metabolism, synaptogenesis, and memory consolidation; and AQP1 and AQP4 adaptations have been seen in obstructive and non-obstructive hydrocephalus and may be therapeutic targets.

## 1. Introduction

Since the discovery of the aquaporin family of proteins, integral membrane pores facilitating diffusion of water molecules, researchers have finally been able to uncover the true nature of physiological osmotic balance. We now have a clearer understanding of the structure, function, and distribution of aquaporins, and begin to discover their contributions to many disease states. Aquaporin molecules are basically involved in water movement in tissues as well as cellular migration and angiogenesis in tumor formation [1], development and resolution of cytotoxic and vasogenic edema [2,3], synaptogenesis and memory formation [4], cerebrovascular disease [5,6], neuroimmunology [7,8], and support of neurostructures of sensory organs [9]. The ubiquity of aquaporin channels in the nervous system might provide many targets for novel therapies for diseases from brain ischemia to traumatic brain injury to Alzheimer’s disease. The contributions of aquaporins to various pathophysiologies is predicated on their ability to mediate the entry and exit of water from the central nervous system. The permeability of the central nervous system is dependent on the distribution, number, and permeability of channels; any potential pharmacological interventions will likely focus on the permeability of select populations.

## 2. Structure and Function of Aquaporins

All mammalian plasma membranes are permeable to water, but with great variability. Those that are only marginally permeable to water are believed to only experience diffusion of water molecules across the lipid bilayer. Those tissues that are more permeable—such as the renal epithelium and erythrocytes—are granted this property by aquaporins, homotetrameric proteins present in essentially all organisms, with 13 discrete forms in mammals. Aquaporins are members of a family of integral membrane proteins, which form pores that allow for the passage of water through membranes, while blocking passage of ions and charged solutes. A subset, termed aquaglyceroporins, can transport small, uncharged molecules as well, such as glycerol and ammonia. While movement of water across the plasma membrane occurs by both passive diffusion and channel-mediated transport, the velocity of flow is much faster through aquaporins [10]. Thus, aquaporins regulate the temporal profile of water movement allowing for more water molecules to be transferred per unit of time, compared to diffusion alone, and obey the rules of osmosis.

Aquaporins were discovered coincidentally as a contaminant while attempting to isolate Rh polypeptides. This contaminant, initially named CHIP28, which stands for channel-forming integral protein of 28 kDa, was found to be a tetrameric integral membrane protein, expressed in high concentrations in erythrocytes and cells of the renal proximal tubule. The presence of a water channel in erythrocytes was suggested by Benga et al. in 1986 [11]. Experiments in Xenopus oocytes revealed that cells expressing CHIP28 swelled in response to a hypotonic buffer, lysed when placed in distilled water, but saw no detectable movement of any solute. These results strongly suggested CHIP28 functioned as a water channel [12]. The discovery and subsequent research on aquaporins earned Agre and colleagues the Nobel Prize in Chemistry in 2003.

Aquaporins contain six transmembrane alpha helices, with two highly conserved loops, each with a characteristic motif, asparagine-proline-alanine (NPA). These NPA loops—cytoplasmic portion B and extracellular portion E—are oriented at 180 degrees with respect to one another [13]. Experiments suggest that the B and E loops form an “hourglass” structure, with the two chambers connecting to form the aqueous pore [14]. The shape of the aquaporin channel core allows only the passage of one water molecule per time, acting as a filter.

Many initial discoveries about the function of aquaporins were made in the kidney, where they are vital for water reabsorption. Immunohistochemical studies have localized AQP1 in the apical and basolateral membranes of the proximal tubule and descending thin limbs of Henle [15] and the descending vasa recta [16]. The AQP1 was identified at the plasma membrane, but not any intracellular locations, nor was it located in the collecting duct. Nielsen and colleagues suggested that water is transported across the renal epithelium through apical and basolateral plasma membranes, driven by the standing osmotic gradients established by movement of solutes through these membranes [17]. AQP1 has also been identified in other secretory tissues, including cholangiocytes, non-pigmented epithelium in the anterior compartment of the eye, and the choroid plexus [18].

### Aquaporin Distribution in the Central Nervous System (CNS)

There are 8 aquaporins expressed in the CNS: AQP1, AQP3, AQP4, AQP5, AQP7, AQP8, AQP9, AQP11, with AQP1 and especially AQP4 expressed in the highest concentrations [19,20,21,22]. AQP1 and AQP4 are structurally and functionally similar, except that unlike AQP1, AQP4 is not sensitive to mercurials [23]. This property is derived from the absence of a cysteine preceding the NPA motif of loop E [24].

AQP1 is expressed at the apical and basolateral surfaces of the choroid plexus and functions in the production of cerebrospinal fluid (CSF). Its greater presence on the apical membrane underlies its role in transcellular movement of water for the production of CSF [18,25]. AQP3 was found in the piriform cortex, hippocampus, dorsal thalamus, globus pallidus (GP), and choroid plexus and also at the border region of ischemic stroke in rats [26]. AQP4 is pervasive throughout the brain and retina, most prominently in astroglia at brain-liquid interfaces. End feet membranes adjacent to the ventricles, capillaries, and subarachnoid space contain 10–15 times the number of AQP4 proteins compared to non-end feet membranes, with microvilli expressing no AQP4 at all [19]. AQP4 is also found in the hippocampal dentate gyrus and Cornu Ammonis (CA) areas CA-1 and CA-3, nucleus of stria terminalis, medial habenular nucleus, neocortex, cerebellum, and supraoptic and suprachiasmatic nucleus of the hypothalamus [27]. AQP5 was localized on the cytoplasmic membrane and in the cytoplasm of astrocytes and was found to be expressed when the brain is exposed to metabolic stress such as ischemic or traumatic injuries [28,29]. AQP8 was also found in the pyriform cortex, hippocampus, dorsal thalamus and globus pallidus and it’s expression was correlated with the grade of astrocytomas [30]. AQP9 belongs to the aquaglyceroporin family, and is permeable to water as well as neutral solutes such as glycerol, lactate, and urea. AQP9 is expressed in astrocytes, cerebellar neurons, pial vessel endothelium, glia limitans, hypothalamic tanycytes, and CA2 of the hippocampus [20,27]. The physiological relevance of AQP7 and AQP11 has not yet been elucidated, although recent literature shows that AQP11 is localized at the epithelium of the choroid plexus and at the endothelium of the brain capillary, suggesting potential involvement in water transport at the choroid plexus and blood-brain barrier (BBB) in the brain. Brains of AQP11-deficient mice, however, did not show any morphological abnormalities and the function of the BBB was intact. AQP11 was expressed mainly in the pia matter with limited expression in the capillary at early postnatal stages. It is possible that AQP11 is needed by the developing brain, to support the water flow [31].

## 3. Role of Aquaporins in Cerebral Edema

Cerebral edema is associated with many neurological disorders, including ischemic injury, traumatic brain injury (TBI), and brain tumor, ultimately leading to increased intracranial pressure and its associated comorbidities, such as ischemia, brain herniation, and death. Under normal conditions, water moves bidirectionally across the BBB, obeying the rules of osmosis, into the CNS and away via the CSF and venous circulation. Three types of brain edema have been described per Klatzo and modified by Fishman [32]: cytotoxic (cellular), vasogenic and interstitial (or hydrocephalic). In cytotoxic brain edema, perturbation of cellular metabolism promotes excess fluid movement across an intact BBB and contributes to an enlarged intracellular space; in vasogenic brain edema, a dysfunctional BBB allows for macromolecules and water to accumulate in an enlarged extracellular space [3]. Interstitial edema occurs in obstructive hydrocephalus, resulting in increased movement of brain water and Na+ across the periventricular walls [32]. Aquaporins line the periventricular wall. Therefore, edema can be viewed as a dysfunction of aquaporins to properly prevent or facilitate water movement. Different types of AQP4 expression can be found in Table 1.

**Figure 1:**
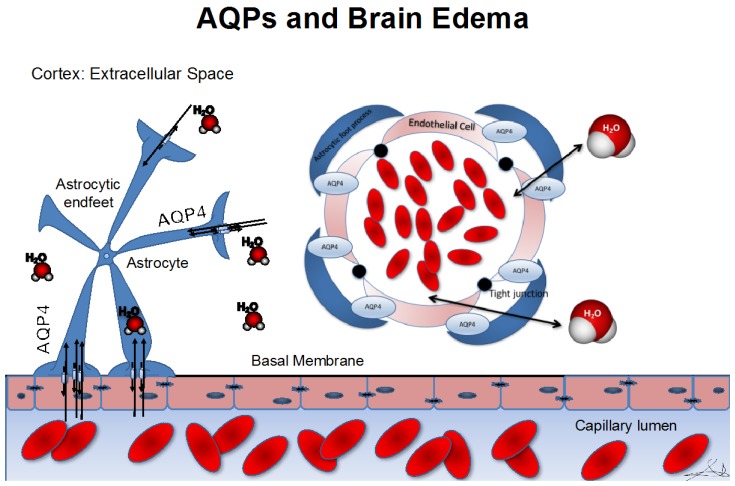
AQP4 distribution in relation to astrocytic feet processes. On the left hand side of the picture, a sagittal slice of brain extracellular space is depicted together with wrapping of the astrocytic endfeet around the endothelium; On the right hand side, the same principle is demonstrated with a coronal dissection of the vessel. Swelling of the astrocytic endfeet after influx of water through AQP4 water channels is characteristic in cytotoxic edema.

Experiments in mice have shown improved survival and neurological outcomes in response to cytotoxic edema following pharmacological inhibition of AQP4 [2]. Reduced intracranial pressure was also observed in AQP4-null mice in cytotoxic edema mimicking meningitis [34]. AQP4 inhibition perturbs its ability to mediate water influx into the astrocytic end-feet and across the BBB [33], disrupting the cascade of events and leads to increased cerebral fluid volume, elevated intracranial pressure, and death. Of note, AQP4-null mice show no profound neurological deficiencies as baseline. It seems that only if AQP4 is present in mice it makes cytotoxic edema worse in pathologies associated with cytotoxic edema (Figure 1).

Experiments have indicated that brain edema is a primarily cellular process. It is believed that protein kinase C (PKC) downregulates AQP4 expression at the astrocytic end-foot process by decreasing AQP4 mRNA [41]. Treatment with phorbol 12-myristate 13-acetate (PMA), an irreversible activator of PKC, reduces cerebral edema concurrently with AQP4 expression in astrocytic end-foot processes in a dose-dependent manner [42]. This supports the hypothesis that edema occurs at least in part due to AQP4 overexpression in the astrocyte following CNS injury.

AQP4 molecules are tethered to the cell membrane by α-syntrophin, dystrophin, and other proteins, and are vital for the localization of AQP4 to the astrocytic end-feet. Experiments in α-syntrophin knockout mice show basal swelling of astrocytic end-foot processes, paralleled with decreased expression of AQP4 at the membrane. Cerebral ischemia also produced less brain edema compared to wild-type [43].

Astrogliosis occurs following CNS injury, and involves synthesis of glial fibrillary acidic protein (GFAP) and reactions by astrocytes [44]. Quantitative measurements of GFAP, indicative of astrogliosis, help define the severity of CNS injury [45]. Recent evidence has linked CNS injury and astrogliosis to increased concentrations of GFAP, AQP4, and Vasopressin 1a receptors (V1aR), the primary arginine vasopressin (AVP) receptor subtype of the CNS [19,46]. Elevations in AVP are witnessed in CSF and serum following TBI [47]. V1aR expression on astrocytes may aid in the movement of water across the astrocytic membrane, a potential mechanistic explanation of symptoms associated with cytotoxic edema [48]. While the exact mechanism by which this occurs is unclear, inhibition of V1aR has been shown to both reduce brain edema [48] and modulate astrocyte swelling and the expression of GFAP and AQP4 [49,50,51], strongly suggesting that V1aR and AQP4 expression mediate cytotoxic edema.

Despite these discoveries, hyperosmotic therapy or surgical decompression remain the two primary treatment options for brain edema in traumatic brain injury, with few pharmacological options available [52,53]. However, inhibition of AVP action in the brain following cytotoxic edema may present itself as an effective clinical tool for decreasing brain edema [50].

Yet, while AQP4-null mice respond better to cytotoxic edema, where the pathology is predicated on water movement through AQP4, they are more susceptible to complications following vasogenic edema. Astrocytic end-feet are pivotal components of the BBB, and regulation of water movement across the BBB is thus heavily dependent on AQP4. In vasogenic edema, where water accumulates in the brain due to leaky vessels, evidence supports AQP4 expression contributes to the enlarged extracellular fluid space [3]. This suggests an additional role for AQP4, as extracellular fluid was believed to move out of the CNS by bulk flow independently of intracellular transport mechanisms.

### 3.1. Hypoxia-Induced Changes in Aquaporin Expression

During early ischemia, anaerobic glycolysis and leakage of lactic acid from necrotic tissue causes acidosis, where protons are exchanged for extracellular Na^+^ by Na^+^/H^+^. Cl^−^/HCO_3_^−^ antiporters played also significant role [54]. Increased glutamate release from necrotic tissues is also transported via the Na^+^ gradient [55]. To counteract the influx of osmoles, glial cells concurrently intake water to compensate for the increased intracellular osmolarity, a process primarily mediated by AQP4.

The exact role of AQP4 in cases of ischemic injury is unclear. In hypoxia-induced brain edema, cytotoxic edema occurs first, followed by vasogenic edema. AQP4 expression has been shown to both decrease in the 48 h following hypoxia [56,57] and increase, although on a slower time scale [58,59]. Taniguchi and colleagues described a biphasic expression pattern of AQP4, peaking at hours 1 and 48h following ischemic injury. These two time points may correspond to focal ischemia and vasogenic edema, respectively. These results are consistent with others suggesting that the hypoxia inducible factor (HIF)-binding motif is found within the AQP4 promoter, and that HIF-1α upregulates AQP4 expression following hypoxic injury [58].

### 3.2. Traumatic Brain Injury

Brain edema is a common complication of TBI, with vasogenic edema occurring rapidly after the injury, primarily in the center of the lesion, while cytotoxic edema has a later onset with a more pervasive effect [35,60]. In traumatic brain injury, in humans, Marmarou et al. demonstrated with MRI studies, that cytotoxic (cellular) brain edema is predominant [61]. Based on this information we could possibly explain why most of the studies in TBI demonstrate an increase in AQP4 expression post-injury, and also why studies or treatments that result in AQP4 reduction/inhibition in these settings could possibly reduce brain edema. Expression levels of AQP4 mRNA have been shown to be elevated in the injured area compared to other brain areas, and the degree of expression correlates to the severity of cerebral edema as measured by MRI. However, tissues immediately adjacent to the affected areas showed downregulation of AQP4, potentially as a protective mechanism [62,63].

### 3.3. Edematous Brain Tumors

Both benign and malignant brain tumors produce brain edema which could be due to defective tight junctions between endothelial cells of the BBB, and increased angiogenesis within the tumor itself [64]. While production of brain edema in tumors is independent of AQP4, expression is upregulated in edematous brain tumors and does not exclusively localize to astrocytic end-feet in these tumors [36]. AQP4-null mice with edematous brain tumors showed increased intracranial pressure and more neurological complications compared to wild-type, indicating AQP4 may have a protective effect in this case [36]. However, this contrasts with studies showing that AQP1 and AQP4 have been shown to promote cancer metastasis through their role in endothelial cell migration and angiogenesis [39]. Reports also imply that upregulation of the AQ4 M1 orthogonal array structure might be responsible for the loss of the normal orthogonal array structure, a structure necessary for AQP4 to maintain it’s function [65]. AQP contribution in gliomas is multiparametric as described by Dubois et al. and Maugeri et al. [66,67]. We mentioned previously that AQP8 in human astrocytomas is associated with the pathological grade, with higher expression in higher grades. AQP9, is highly expressed in tumor stem cells, with resistance to treatment [66,67].

## 4. Hydrocephalus

Hydrocephalus was described by the Rekate model as the disease state that evolves after obstruction of CSF circulation. The points of obstruction could be in different sites and areas in the macro-world (e.g., aqueduct of Sylvius) or micro-world (e.g., channels) [68]. Given the role of aquaporins in both production (via AQP1) and absorption of CSF (via AQP4), they are a potential therapeutic target. AQP1-null mice had osmotic water permeability reduced by a factor of five compared to wild-type, reduced CSF production by 20%–25%, and intracranial pressure by 56%. While much of this effect was due to decreased central venous pressure due to effects of AQP1 deficiency in the kidney, the results suggest reducing AQP1 function decreases both production of CSF and development of nonobstructive hydrocephalus [69,70].

Smith and colleagues describe the case of a 15-month-old girl with choroid plexus hyperplasia, leading to CSF overproduction and nonobstructive hydrocephalus. Immunohistochemical analysis showed decreased AQP1 expression in samples taken in the patient compared to controls, which is indicative of adaptive downregulation [37]. However, further studies have indicated a more heterogenous expression of AQP1 in cases of hydrocephalus, but still supporting an adaptive mechanism that decreases AQP1 expression [71,72].

AQP4 appears to have a protective role in cases of hydrocephalus, given its role in CSF absorption in cerebral vasculature. AQP4-null mice showed significant ventriculomegaly after kaolin injection to reproduce obstructive hydrocephalus, increasing intracranial pressure by 2%–3%. After 5 days, the mortality of AQP4-deficient mice was 34% compared to 16% of wild-type [73]. After kaolin-induced hydrocephalus in wild-type mice, AQP4 expression was increased 3–4 weeks post-injection, with highest levels in the perivascular areas, parietal cerebrum and hippocampus, ependymal lining, and glia limitans [74]. Similar results were seen in expression patterns at the blood-CSF barrier and BBB [38].

## 5. Role of Aquaporins in Cellular Migration

In response to trauma, reactive glial cells produce scar tissue; scar formation facilitates repair of the BBB, minimizes neuronal death, and prevents migration of inflammatory cells to the site of injury. However, scar formation limits the brain’s capacity for axonal regeneration. This process is believed to be mediated at least in part by AQP4. Astrocyte migration and formation of scar tissue were delayed in AQP4-null mice, and blockade of AQP4 may be a novel method of promoting synaptogenesis and axonal sprouting following CNS injury [40,75]. Saadoun and colleagues propose that AQP4 mediates an influx of water to dilute the accumulation of ions and depolymerized actin at the leading edge of astrocytes. The membrane of the leading edge is stretched by the increased intracellular hydrostatic pressure in the direction of the leading edge. The increased space provided permits actin repolymerization, thereby promoting cellular migration.

Proliferating microvessels of malignant tumors have been shown to highly express AQP1, as well as some tumor cells. Blockade of AQP1 expression has been shown to decrease angiogenesis and induce tumor necrosis [1,39,76]. AQP1 expression is vital for cellular migration in endothelial cells: AQP1-null endothelial cells show slowed, impaired migration towards chemoattractants compared to wild-type [77]. Both AQP1 and nitric oxide (NO) levels are elevated during brain edema. NO production by nitric oxide synthase (NOS) confers a protective function, promoting vasodilation of ischemic areas. Experiments blocking endothelial NOS (eNOS) also caused a parallel decrease in AQP1 expression, indicating that the two are linked by a common signaling pathway [78].

AQP1 and AQP4 are both potential targets to modulate migration: when expressed in CHO or FRT epithelial cells, both aquaporins lead to enhanced cellular migration compared to AQP-null. Video microscopy studies have shown that AQP1 facilitates the growth of more lamellipodia and a decreased mean residence time, indicating AQP1 assists in the rapid turnover of lamellipodia at the leading edge [77].

## 6. Epilepsy, Memory Consolidation, and Aquaporins

Epilepsy is a medical condition characterized by unpredictable onset of seizures. Approximately 30% of patients who take antiepileptic drugs do not see a complete reversal of symptoms, and current antiepileptic drugs are accompanied by a host of developmental, behavioral, and cognitive side effects, since they target neurons directly [79]. While neuronal excitability is pivotal to the hyperexcitability of epilepsy, evidence supporting astrocytic involvement is growing and could be an avenue for potential therapeutics.

Glial cells are of paramount importance to electrophysiological function of neurons, providing energy and nutrition, recycling neurotransmitters, regulating ionic homeostasis, and modulating synaptogenesis, and are believed to be integrally involved in many cases of epilepsy [43,80]. Hypoosmolarity and decreased extracellular fluid volume in the brain induces hyperexcitability and seizures, and the extracellular environment is modulated primarily by astrocytes [81]. AQP4-null mice have a decreased extracellular space (ECS) compared to wild-type; this corresponds to less seizure activity after administration of pentylenetetrazol (PTZ), a GABA_A_ antagonist [82]. They do, however, exhibit a longer seizure duration than that of wild-type [83].

Both AQP4 and K_ir_4.1, an inwardly rectifying potassium channel, are colocalized to the perisynaptic region of the astrocytic endfoot [84]. Increased neuronal activity increases extracellular [K^+^] substantially, most of which is taken up by neighboring astrocytes, mediated in part by K_ir_4.1; inactivation of K_ir_4.1 hampers regulation of extracellular K^+^, and thus disrupts potassium siphoning, a significant mechanism of extracellular potassium clearance [84]. Levels of K^+^ concentration, [K^+^], after baseline and moderate activity were not drastically affected by AQP4 deficiency, with heavy stimulation-induced elevations showing slower time kinetics compared to wild-type. However, K^+^ kinetics after stimulation are delayed in AQP4-null mice, both in rise and decay, indicating a role of AQP4 in regulating K^+^ homeostasis and neuronal activity [83]. These results are consistent with previous studies showing a longer duration of seizure in AQP4-null mice, as prolonged depolarization would block seizure termination.

AQP4 is tethered to the membrane by α-syntrophin. Deficiencies in α-syntrophin disrupt AQP4 expression at the perivascular and subpial membranes [43], and dystrophin deficiencies produce lower levels of expression at astrocytic end-foot processes, the polarized expression of AQP4 is impaired. despite normal AQP4 protein levels [85]. Research in mice has shown α-syntrophin-deficient mice exhibit greater seizure intensity in response to hyperthermia, coupled to a decrease in extracellular K^+^ clearance, due to decreased potassium siphoning [43]. While it is clear that AQP4, K_ir_4.1, and associated anchoring proteins form a multifunctional unit involved in K^+^ homeostasis, the exact mechanism behind their activity remains unclear [86].

### Glutamate Metabolism and Synaptic Plasticity

Low extracellular glutamate levels must be maintained in the ECS to ensure proper neuronal excitability, mediated by glutamate transporters, most prominently glutamate transporter-1 (GLT1) [87]. GLT1 deficiencies produce spontaneously epileptic activity due to overabundance of glutamate, while overexpression of GLT1 reduces seizure frequency [88,89].

Prolonged status epilepticus (SE) induces a downregulation of AQP4 in mouse hippocampal astrocytes at day 1 post SE with a prolonged period of recovery, and concurrent upregulation of GLT1 at day 1 with significant decrease in expression levels at days 4 and 7 post SE. These results were present even in the absence of cell death or sclerosis, indicating that profound seizures regulate AQP4, GLT1 expression [90]. Downregulation of AQP4 may lead to both hyperactivity and dysfunctional synaptic plasticity and cognition, both of which are significant comorbidities of epilepsy.

The hypothesized role of AQP4 is consistent with results showing dysfunctional synaptic plasticity. AQP4-null mice exhibited deficiencies in long term potentiation (LTP) and long term depression (LTD) of the hippocampus, without significantly affecting synaptic transmutation or short-term plasticity [91]. These findings were supported by others showing that AQP4-null mice exhibited impaired memory consolidation as measured by the Morris water maze (MWM) [92]. Similar experiments in the amygdala of AQP4-null mice showed impaired LTP and decreased associative fear memory, as well as downregulated GLT1 expression, further suggesting that AQP4 has a pivotal role in memory consolidation [93].

The role of GLT1 as the mediator of AQP4-dependent memory deficiencies was supported by experiments showing that LTP and memory formation were rescued in AQP4-null mice when treated with ceftriaxone, a stimulator of GLT1 [94]. Accumulations of glutamate, resultant of reduced GLT1 expression in AQP4-deficient mice, causes strong activation of NMDA receptor (NMDAR) [95], and increased NMDAR-mediated currents were seen in mice lacking AQP4. Pharmacological inhibition of NMDAR rescued LTP dysfunction in AQP4-deficient mice, suggesting excess glutamate in the synaptic cleft perturbs NMDAR-mediated currents and LTP in AQP4-deficient mice [93].

## 7. Aquaporins and the Glymphatic System

Aside from recent discoveries of lymphatic vessels in the dural sinuses [96], the brain is believed to lack the traditional lymphatic structure of the rest of the body and removes interstitial fluid (ISF) differently. Instead, the brain utilizes the glymphatic system, a microscopic fluid clearance system formed by astrocytic perivascular tunnels that remove fluid, proteins, and waste molecules, and distributes compounds such as nutrients, growth factors, and neuromodulators. In this system, the ISF and CSF constantly exchange, with movement of CSF into the brain parenchyma facilitated by AQP4 in the astrocytic end-feet [97].

Iliff and colleagues used fluorescent tracers to show CSF enters the brain parenchyma via periarterial pathway surrounding the smooth muscle cells adjacent to astrocytic endfeet, and exited the brain via central deep veins and the lateral-ventral caudal rhinal veins. In many neurodegenerative diseases, there is an accumulation of proteins, contributing to disease pathology. It was discovered that amyloid-β was cleared along the glymphatic pathway, and AQP4-null mice showed a reduction in CSF flux of 65%, and a reduction in amyloid-β clearance of 55%, suggesting a role of AQP4 in neurodegenerative diseases such as Alzheimer’s [97,98]. Antoine Louveau et al. and Aspelund et al. [96], while searching for T-cell gateways in the brain, pinpointed the presence of functional lymphatic vessels lining the dural sinus and draining to the deep cervical lymph node complex. This pathway, which was not brought to light until recently, might be responsible for an alternative approach to current neuroimmunology and dogma that govern the immune response of the brain to disease insults. This dural lymphatic vascular system is capable of draining brain interstitial fluid and macromolecules as both Louveau et al. and Aspelund et al. report [96].

## 8. Looking Forward

Aquaporins definitively represent a subset of channels that significantly contribute to the physiology of cells. Their ability to facilitate water movement both in and out of the CNS contributes greatly to brain water homeostasis. To be able to utilize their contributions therapeutically, we need to identify non-toxic molecules to act as AQP inhibitors or amplifiers based on the expected benefit in each case. A discovery, by error, shows a vast potential to alter the “roux” of history for the future of the theraupeutic modulation of brain edema. Our mission is to more deeply understand the physiology of aquaporins in neuroscience and identify their modulators.

## Figures and Tables

**Table 1:** Expression of AQP4 in different pathologies.

Process	Species	AQPs Studied	Results
Cytotoxic edema [33]	Mice	AQP4	Upregulated
Vasogenic edema [34]	Mice	AQP4	Upregulated
Traumatic brain injury [35]	Rats	AQP4	Upregulated
Edematous brain tumor [36]	Human	AQP4	Upregulated
Hydrocephalus [37,38]	Human	AQP1	Downregulated
	Rat	AQP4	Upregulated
Cellular migration [39,40]	Mice	AQP1	Upregulated
	Mice	AQP4	Upregulated
Prolonged status epilepticus [4]	Mice	AQP4	Downregulated
